# Harnessing the Microbiome to Enhance Cancer Immunotherapy

**DOI:** 10.1155/2015/368736

**Published:** 2015-05-25

**Authors:** Michelle H. Nelson, Marshall A. Diven, Logan W. Huff, Chrystal M. Paulos

**Affiliations:** ^1^Department of Microbiology and Immunology, Medical University of South Carolina, Charleston, SC 29425, USA; ^2^Department of Surgery, Medical University of South Carolina, Charleston, SC 29425, USA

## Abstract

The microbiota plays a key role in regulating the innate and adaptive immune system. Herein, we review the immunological aspects of the microbiota in tumor immunity in mice and man, with a focus on toll-like receptor (TLR) agonists, vaccines, checkpoint modulators, chemotherapy, and adoptive T cell transfer (ACT) therapies. We propose innovative treatments that may safely harness the microbiota to enhance T cell-based therapies in cancer patients. Finally, we highlight recent developments in tumor immunotherapy, particularly novel ways to modulate the microbiome and memory T cell responses to human malignancies.

## 1. Introduction

Mammals have over 100 trillion microbes in distinct locations of the body-outnumbering mammalian cells 10-fold. Nearly 1000 different types of microbes colonize the host. Moreover, healthy individuals differ vastly in the type of microbes that colonize their gut, likely as a consequence of their exposure to microorganisms after birth, genetics, environmental cues, and diet. These diverse microbial communities are collectively referred to as* the microbiota* [1]. Beyond aiding in digestion and nutrient acquisition, microbes impact health and disease via regulating the immune system [2].

Mutualistic microbes that colonize the gut are crucial for health. These microbes sustain basic physiological processes—digestion, vitamin synthesis, and host-defense [3–5]. However, disruption of this homeostatic host-microbe relationship can promote disease pathogenesis, such as various autoimmune diseases [6–8]. Changes in the microbiota can also influence tumor immunity. As cancer therapy develops, it is vital to understand the impact of these treatments on host-microbes and the immune system [9].

## 2. Coley's Toxin in Tumor Immunotherapy

In the late 19th century Coley treated human malignancy with live bacterial cultures [10, 11]. He suspected that erysipelas could treat sarcomas based on 90 surgical cases at the New York Hospital [12]. One patient experienced a complete regression of neck sarcoma and metastasis after infections with erysipelas. Inspired by this case, he injected live streptococcal organisms into another patient with an inoperable sarcoma. This patient experienced durable antitumor responses. Coley proceeded to create a safer bacterial concoction comprised of heat inactivated streptococcal organisms along with* Serratia marcescens*, known later as Coley's toxin. He treated nearly 1,000 sarcoma patients [13]. A projected 80% of these patients experienced an increase in survival of up to 5 years. This was an impressive result for a disease where there existed no effective treatment [14, 15]. Coley hypothesized that the toxins within the killed bacteria induced durable immune responses in patients. Over the years, researchers have obtained data to support, as well as to negate, this idea [16–19]. The goal to harness the patient's immune system to kill tumors remains a major goal in the field of cancer immunotherapy [20–22].

## 3. Commensal Microflora Regulates the Innate and Adaptive Immune System

There has been significant advancement in our understanding of the microbiome in health and disease since Coley's toxin. It is now well appreciated that a diverse population of bacteria, Archaea, Eukaryota, and viruses colonize the gut [23]. This colonization process begins at birth and stabilizes around the age of three years [24, 25]. The microbial composition impacts the immune system and in turn regulates the microbiome [24]. Nutrition, therapeutic interventions, and age are factors that influence the type of bacteria in the gut [26, 27]. Emerging data reveal that antibiotics can detrimentally regulate microflora [28, 29] and thus impair immunity, by exacerbating autoimmunity, dampening antitumor responses, and increasing mortality in patients receiving organ transplants [6, 30]. Consequently, when feasible, narrow spectrum antibiotics are prescribed to minimize the risk of altering the composition of the microbiome [31].

Intestinal epithelia maintain a physical barrier between gut microbiota and the internal cavity. Overexposure to antigens causes improper gut immune function. For example, gut bacteria enhance the capacity of mucosal B cells to produce IgA and IgM antibodies [32]. In the absence of IgA, anaerobic bacteria expand; this includes segmented filamentous bacteria (SFB). Recently, SFB were found to support the generation of inflammatory CD4^+^ T cells that secrete IL-17, called Th17 cells [33]. Additional investigation revealed that SFB-induced Th17 cells exacerbate autoimmunity, such as arthritis, that could be tempered by treating mice with certain antibiotics that reduced Th17 cells. Based on such interesting data, other investigators became motivated to target the microbiome to enhance Th17 and CD8^+^ T cell responses against tumors. After reviewing TLRs in cancer immunotherapy (immediately below), we will then discuss various strategies for manipulating the microbiome to bolster T cell-mediated tumor immunity, including chemotherapy, cellular therapy, and antibiotics.

## 4. Toll-Like Receptors in Cancer Immunotherapy

The hosts' innate immune system senses microbes via various pattern recognition receptors (PRRs). These PRRs recognize microbes and activate dendritic cells (DCs) [34]. Once PRRs recognize pattern-associated molecular patterns (PAMPs), a cascade of intracellular signaling pathways is triggered, resulting in the activation of immune cells [35, 36]. The innate immune response is the first line of defense against microbial infection and triggers an effective adaptive immune response. Different PRR agonists can synergize to induce a broad immune response [37]. For centuries, these agonists have been used as vaccines to treat cancer patients, albeit with limited success.

The most studied PRRs are toll-like receptors (TLRs) and are conserved between plants, insects, and mammals. There are 10 known functional human TLRs that detect PAMPs from fungi, bacteria, viruses, and protozoa [35]. TLRs link the innate and adaptive immune system by recognizing pathogens via DCs and in turn boosting T and B cell responses. Synthetic TLR agonists induce immune response without the toxic side effects induced by whole pathogens. These agonists have been used to enhance cancer vaccine. Some agonists are discussed below and are outlined in Table 1.

### 4.1. TLR3 Agonists

Double-stranded RNA (dsRNA), such as polyinosinic:polycytidylic acid (Poly(I:C)), triggers TLR3 on DCs and has been used as an adjuvant to enhance immune responses [38, 39]. One synthetic Poly(I:C) molecule called Ampligen was reported to promote DC maturation, type I immune responses, and tumor regression in mice [40]. A degradation resistant poly-ICLC molecule called Hiltonol was also found to mediate anticancer responses in primates. Both molecules are being tested alone or in combination with other adjuvants (such as NY-ESO-1 antigen or DCs) in ongoing phase I and/or phase II cancer clinical trials (http://www.clinicaltrials.gov/).

### 4.2. TLR4 Agonists

LPS is a component of the outer membrane of Gram-negative bacteria and triggers TLR4 [41]. Numerous studies show that LPS, used as a single agent or in combination with other therapies, can induce antitumor immunity in mice [6, 42–44]. However, even small quantities of LPS induce toxic shock in cancer patients by triggering a cytokine storm by immune cells [45]. Consequently, LPS was modified to separate the immunomodulatory from the unwanted toxic side effects of the parent molecule. This modification generated monophosphoryl lipid A (MLA), as well as other nontoxic derivative molecules (such as MPL, AS04, and GLA-SE). TLR4 agonists have been used alone or in combination with other therapies in humans yet have only mediated minor successes as a cancer adjuvant. Nonetheless, Picibanil (OK-432), a preparation of* Streptococcus pyogenes* which triggers TLR4 signaling, has been approved for clinical use and is used in Japan to treat patients with various carcinomas [46, 47].

### 4.3. TLR5 Agonist

Flagellin is the only known natural ligand for TLR5. This agonist has clinical promise, as the peptide derivative of* Salmonella enterica* (CBLB502) was found to protect animals from high dose radiotherapy [48, 49].

### 4.4. TLR7/8 Agonists

TLR7 and TLR8 are located in the endosomal compartment and are stimulated by small synthetic compounds and natural guanosine- (G-) and uridine- (U-) rich single stranded nucleosides that characterize viral RNA [50–52]. Numerous trials are ongoing using imiquimod (TLR7) or resiquimod (TLR7/8) as a single agent or in combination with other vaccines. Imiquimod (Aldara) is FDA approved and used to treat patients with melanoma and VTX-2337 (a TLR8 agonist) has been used in phase II clinical studies to treat patients with head and neck squamous cell carcinoma (HNSCC) as well as cancers of the reproductive tract and peritoneal cavity. These various TLR7/8-based trials can be found at http://www.clinicaltrials.gov/.

### 4.5. TLR9 Agonist

Species-specific sequences of unmethylated deoxycytosine-deoxyguanosine (CpG) motifs from bacterial and viral DNA stimulate TLR9. A variety of CpG derivations have been tested clinically and are nontoxic, but their effectiveness is modest. In many studies, these adjuvants boosted immune responses but do not drive tumor regression or prolonged survival in cancer patients [53, 54].

## 5. TLR Expression on T Cells and Cancer Cells

Studies have long focused on the role of TLR signaling on antigen presenting cells (APCs) and how this signaling shapes the adaptive immune system. However, T cells also express functional TLRs, which can influence their fate. Although TLRs are expressed at lower levels on T cells than on APCs, TLR agonists can directly activate T cells [55, 56]. Moreover, DC stimulation via specific TLRs (i.e., TLR3, TLR7, and TLR9) endows them with the enhanced ability to present antigen, leading to antigen-specific T cell activation [56, 57]. TLR signaling augments CD8^+^ T cells function, as demonstrated by their heightened capacity to simultaneously secrete IFN-*γ*, TNF-*α*, and IL-2 [58, 59].

Regulatory T cells (Tregs cells) express TLRs [60] and triggering them with agonists can either enhance or dampen their suppressive function [61, 62]. The ability to regulate Treg-induced suppression via TLR agonists affords new opportunities for designing vaccines. Thus, researchers have worked hard to identify TLR agonists that preferentially trigger DCs and effector T cells, but not Treg cells, to treat cancer patients.

Finally, tumor cells also express TLRs and triggering TLR ligation promotes their expansion, invasion, and metastasis [63]. TLR therapy can have direct and indirect tumoricidal effects. As such, inflammation was added to Hanahan and Weinberg's “Hallmarks of cancer” model [64]. It is understood that cancer cell development is associated with inflammatory signals induced by microbial infection. Part of this inflammatory equation is due to the upregulation of TLRs on tumor cells, which activate a series of signaling events to promote tumorigenesis [65]. Therefore, TLR agonists have the potential to positively or negatively affect an antitumor response.

## 6. Vaccines Promote Weak T Cell-Mediated Tumor Regression

Cancer vaccines fail to mediate the regression of large tumors in mice [9]. Similarly disappointing results with vaccines have been reported in cancer patients. Hundreds of clinical trials with vaccines have been conducted with various formulations, ranging from immunization with DCs, recombinant viruses, peptides, proteins, whole cells, or even naked DNA in conjunction with a variety of adjuvants and TLR ligands. Two reviews have examined the results of published clinical trials and could find rare cases of complete responders and an overall objective response rate of less than 3% [66, 67]. Indeed, more potent therapies to kill tumors and prevent recurrence are warranted. Perhaps combining some of the most promising TLR agonists (discussed above) with vaccines that target somatic mutations exclusive to each cancer, checkpoint modulators, and/or T cell therapies will advance the field [68, 69].

A common vaccine strategy for cancer treatment is to administer tumor-specific peptides in combination with a delivery agent to bolster endogenous CD8^+^ T cells. Numerous clinical trials have demonstrated only marginal success in treating patients with gp100 and incomplete Freund's adjuvant (IFA) [70–72]. Hailemichael and coworkers found that IFA (with gp100) sequesters melanoma-specific T cells at the injection site, thereby decreasing the number of cells that migrate to the malignant site and reducing the antitumor response [73]. The CD8^+^ T cells residing at the injection site have prolonged antigen stimulation and became hyporesponsive; in that, they were apoptotic and unable to expand when rechallenged with tumor. The study also found that covax therapy—which entails the CD40-specific antibody, TLR7 agonist imiquimod and IL-2 with IFA plus gp100—did not prevent vaccination-site sequestration. This combination therapy was still unable to rescue the hyporesponsive cells. Conversely, if saline, instead of IFA, was used as a vehicle to deliver gp100 to the mice, T cells were not sequestered at the vaccination-site and were able to home to the tumor and regress melanoma in mice. These studies suggest that how a vaccine is delivered to a patient might be important for harnessing a potent antitumor response.

In 2005, a clinical trial with TLR9 agonists in melanoma patients appeared promising based on striking T cell responses* in vivo*. Speiser et al. combined CpG ODN 7909 (a 24-mer oligodeoxynucleotide containing 3 CpG motifs) with a Melan-A_26–35_/MART-1 peptide and IFA and observed an increase in the number of MART-1-specific T cells greater than 10-fold compared with patients administered with vaccination without CpG [74]. Surprisingly, this heightened immune response did not promote tumor regression in these patients, implicating that MART-1 T cells induced by CpG and vaccination are functionally tolerized or ineffective due to expansion into a terminally differentiated T cell. The MART-1-reactive T cells induced by CpG-based vaccination might be tolerized by Treg cells. Indeed, Speiser and coinvestigators found that Treg cells were elevated in the tumor of CpG-treatment patients [75, 76]. It is also possible that this vaccination strategy induces other suppressor immune elements, such as MDSCs, that impair the antitumor activity of T cells. These data underscore the need to seek potent strategies to mediate tumor regression in patients.

## 7. ACT Therapy: A Promising Cancer Treatment

Adoptive immunotherapy is a promising treatment for patients with advanced malignancies. For more than two centuries, cancer patients have been subjected to various therapeutic approaches, such as vaccine therapies, and until recently many had poor clinical outcomes. The discovery of cancer-specific antigens has allowed for the development of adoptive cell transfer (ACT) therapy [77], that is, using the patients' T cells to target and kill their cancer. The ACT approach, as initially reported in 1988, mediates objective responses in ~30% of patients with metastatic melanoma [78–81]. This approach involves multiple steps: (1) selecting tumor-infiltrating lymphocytes (TILs) from the resected tumor nodules of patients; (2) rapidly expanding TILs for several weeks* ex vivo*; and (3) infusion of cells into the patient in conjunction with bolus high-dose interleukin-2 (IL-2). This strategy has many advantages over passive vaccine treatments. One can administer a large number of naturally occurring TILs with high avidity for tumor antigens [82]. Both CD4^+^ and CD8^+^ T cells are capable of recognizing tumor antigens and both play a role in the antitumor immune responses [83–85]. These cells can be programmed to different subsets [86], activated from their poorly functional state* in vivo* [87] and sorted for optimal function* ex vivo* [88]. After infusion, these cells are capable of massive expansion [89, 90]. Furthermore, infused T cells can traffic to every site in the body, thus allowing for the clearance of tumors even in the brain [91]. Despite these advantages, this treatment triggered objective immune responses in only a minority of patients [79–81]. Consequently, investigators use lymphodepleting preparative regimens to alter the environment for infused cells, a maneuver that has enhanced treatment outcome by creating space for the infused cells and modulating the microbiota.

## 8. Lymphodepletion Augments ACT Therapy

Transfer of* ex vivo* expanded naturally arising or engineered T cells after lymphodepletion by total body irradiation (TBI) and/or chemotherapeutic drugs is a promising treatment for cancer patients [78, 89, 92, 93]. This method augments the* in vivo* function and persistence of infused T cells, thereby increasing objective response rates in patients compared to those treated with ACT therapy alone.

Chemotherapy can be delivered alone or in conjunction with other treatments. These drugs are targeted to dividing cells, which include most cancer cells, but this therapy is also toxic to normal cells in the immune system and digestive tract [94]. The side effects of this therapy can be severe and even fatal. Thus, understanding how chemotherapy drives T cell-mediated tumor destruction and targeting those mechanisms to safely improve therapies are a major goal of the cancer therapy field.

Radiotherapy can be directed to the tumor site or to the whole body. High doses of radiotherapy of up to 12 Gy TBI are potent, given in fractionated doses, and require hematopoietic stem cell (HSC) transplantation [95–97]. Compared to nonmyeloablative chemotherapy with only cyclophosphamide and fludarabine, which mediates an objective response rate of 50%, patients preconditioned with a myeloablative regimen prior to ACT experienced objective response rates of 72% with some curative responses [78].

## 9. Mechanisms of TBI Effectiveness: Sinks, Suppressors, and Microbial Activators

In 1969, Fefer et al. first reported the idea that lymphopenia-induced expansion of T cells bolsters antitumor immunity in mice [98–100]. They found that infusion of lymphocytes in conjunction with chemotherapy could treat mice with lymphomas. North and coworker confirmed that this approach mediates tumor regression in animals with sarcomas in 1980 [101]. In 2002, Dummer and colleagues found that homeostatic expansion of donor CD8^+^ T cells mediates tumor regression in irradiated animals via cytotoxic/IFN-*γ*-mediated mechanisms [102]. Collectively, their work underscores that lymphodepletion potentiates T cell activity in various mouse models of cancer.

**Figure 1:**
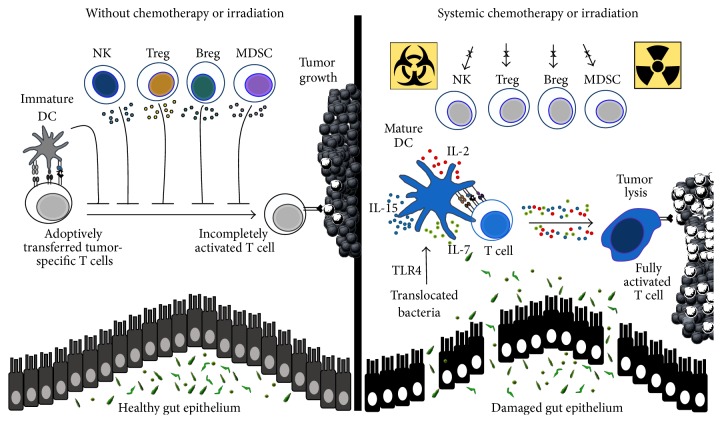
Lymphodepletion enhances the antitumor activity of transferred T cells. Left panel: without chemotherapy, natural killer (NK) cells act as cytokine sinks that compete for homeostatic cytokines (IL-7 and IL-15) that otherwise help transferred T cells engraft. Additionally, immune suppressive cells, such as regulatory B and T cells (Breg and Treg, resp.) and myeloid derived suppressor cells (MDSC), abrogate the function of transferred T cells. Right panel: lymphodepleting preparative regimens eliminate cytokine sinks and immune suppressive cells leading to enhanced function of transferred T cells. Furthermore, systemic chemotherapy or irradiation impairs gut homeostasis leading to the translocation of bacteria and by-products including LPS (TLR4 agonist). Immature dendritic cells (DC) are activated via TLR4 signaling, which in turn activate transferred T cells. Transferred T cells preferentially expand following the consumption of homeostatic cytokines produced by mature DCs, resulting in potent antitumor responses* in vivo*.

Over the past 20 years, the Restifo lab has explored how lymphodepletion augments infused CD8^+^ T cells to kill melanoma in mice and man [103]. Along with creating space, they found that TBI induces microbial translocation in mice, which augments ACT therapy. In an ACT model using pmel-1 CD8^+^ transgenic T cells (specific for the self/tumor antigen gp100), mice were rendered lymphopenic with a nonmyeloablative preparative regimen of 5 Gy TBI. As anticipated, the administration of 5 Gy TBI prior to infusion of pmel-1 CD8^+^ T cells, vaccination, and IL-2 enhanced tumor destruction compared to nonirradiated mice. The mechanisms underlying the effectiveness of lymphodepletion are multifold. As shown in Figure 1, these mechanisms include (1) the depletion of Tregs and MDSCs that limit the function of transferred cells [104–107]; (2) removal of host immune cells (such as NK cells) that act as “sinks” for homeostatic cytokines (IL-7 and IL-15), whose levels are elevated after lymphodepletion [108–111]; and (3) the activation of the innate immune system via TLR4 signaling, which is stimulated by microbial LPS translocated across the gut and by alarmins produced by dying tumor cells [6, 9]. Below, we discuss TBI mechanisms of action and elaborate on how these findings can be exploited to augment T cell therapies.

## 10. Lymphodepletion Activates the Innate Immune System

Total body irradiation increases antigen expression on the cell surface of tumors [112]. Yet, local irradiation does not slow tumor growth in mice [113]. Conversely, systemic 5 Gy TBI before ACT enhanced melanoma destruction even when the tumor was shielded from irradiation. Yet, direct delivery of high dose irradiation to the tumor, up to 20 Gy TBI, does not induce T cell-mediated tumor regression in mice [113]. This work harmonizes with earlier studies by the Hellstrom lab, who first reported that TBI may enhance treatment in mice due to its direct effects on the host not tumor [114].

TBI also compromises the morphological integrity of the gut epithelium, permitting the translocation of microbes into mesenteric LNs and elevated bacterial-derived LPS in the serum (Figure 1) [9]. Bacterial translocation activated the innate immune system, as indicated by the transient activation of host APCs that secreted heighten levels of proinflammatory cytokines in irradiated mice [6, 115–117]. As indicators of DC maturation, the costimulatory molecules CD80, CD86, and CD70, as well as MHC class II, increased on the surface of DCs following TBI [117–120]. Microbe-activated DCs potentiated the function of T cells, thereby mediating cures in mice. Reducing microbial translocation with antibiotic ciprofloxacin decreased the absolute number of activated host DCs in irradiated animals, a consequence that impaired T cell-mediated tumor immunity [9]. Likewise, removal of LPS with polymyxin B reduced the beneficial effects of TBI on tumor regression. Conversely, LPS administration to irradiated animals enhanced the function of the infused T cells, leading to long-term cures of mice with large tumors [9]. Collectively, these data support the notion that microbes regulate antitumor immunity.

## 11. Mimicking TBI: Promoting the Benefits While Removing the Harm

Microbial translocation augments the antitumor activity of adoptively transferred CD8^+^ T cells via TLR4 signaling in irradiated mice [6]. Therefore it is logical to think that exogenous administration of LPS to nonirradiated mice would mimic the benefits of TBI. Yet, this idea was rebuffed, as LPS administration alone did not augment tumor regression in nonirradiated mice infused with melanoma-specific T cells [6]. Thus, innate immune activation with TLR4 agonists cannot replace TBI effectiveness.

Additional work revealed that TBI enhanced adoptive immunotherapy several mechanisms via (1) removal of cytokine sinks, (2) depletion of Treg cells and MDSCs, and (3) microbial activation of the innate immune system. Follow-up work by the Restifo lab revealed that depletion of lymphocytes that act as cytokine sinks alone (via antibody depletion of natural killer cells), removal of regulatory T cells alone (via antibody depletion of CD4 T cells), or activation of the innate immune system via TLR4 signaling alone (via LPS administration 1 day after adoptive cell transfer) could not augment ACT in nonirradiated mice with melanoma [6]. They found that T cell-mediated tumor regression was only achieved by* mimicking all three mechanisms* underlying the effectiveness of TBI. These findings are important because they define key variables needed for TBI to improve cell therapies in cancer patients and sheds light onto how to treat patients that are not ideal candidates for host preconditioning regimens.

Approaches to deplete host lymphocytes that act as suppressors and cytokines sinks have been executed in the clinic, with varying degrees of success. IL-2 receptor (CD25) expressing cells have been depleted in clinical trials with ONTAK, an engineered protein that binds to CD25 and kills cells by exposing them to a fused diphtheria toxin. Since Treg cells express high CD25 levels, this would be an interesting method of limiting these cells in patients. Other studies have tried to deplete suppressive Tregs with HuMax-CD4 (Zanolimumab) and LMB-2 antibodies [121–123]. Approaches that specifically kill Treg cells without removing helper T cells (i.e., Th1 cells) are ongoing goals in the clinic.

B cells, a proportion of which are Bregs, promote breast cancer metastasis in mice [106]. Thus, their ablation with rituximab, a chimeric anti-CD20 monoclonal antibody, might potentiate the antitumor activity of T cells in patients [124, 125]. CD19 CAR T cells have shown promise in treating patients with advanced chronic lymphoid leukemia. Yet, CD19 CAR T cells also kill healthy B cells [92, 126]. While this effect has potentially side effect, it is possible that B cell removal by CD19 CAR T cells helps them thrive* in vivo* and kill leukemia [127]. Mechanisms underlying the effectiveness of CD19 CAR T cells are of interest to the ACT field and how they impact health B cells in tumor immunity are being explored [125].

Furthermore, homeostatic cytokines IL-7 or IL-15 could be administered to bolster the expansion of transferred cells as they are of low basal level in patients. The use of complexes of IL-15 with IL15R*α* (or IL-7 with IL-7R*α* complexes) to boost T cells is shown to be promising in mouse models [128–130]. These cytokine complexes have greater systemic half-life than regular unbound cytokines. The Celis laboratory found that IL-2/anti-IL-2 antibody complexes (when combined with vaccination) could eradicate melanoma in mice receiving tumor-specific CD8^+^ T cells [131]. Thus, delivery of cytokine complexes in patients might potentiate the antitumor activity of infused T cells.

In combination with ACT therapy and reagents that deplete suppressors and sinks, the patient's innate immune system might be safely activated with clinically relevant TLR agonists, including CpG ODN 7909 [9]. The costimulatory molecule CD40 is upregulated on innate immune cells upon activation. Thus targeting CD40 might also enhance cellular therapy through engaging the CD40L molecule on transferred tumor-specific T cells. Beatty and coworkers found that CD40 agonists alter tumor stroma and show efficacy against pancreatic carcinoma in mice and humans [132]. Thus, adoptively transferred CAR engineered human T cells that are specific for pancreatic tumors, along with a CD40 agonist, might elicit tumor regression in patients [133]. Likewise, blockade of coinhibitory molecules PD-1 and CTLA-4 has shown promise in patients with melanoma, renal cancer, triple negative breast cancer, and non-small-cell lung cancer [134, 135] and thus might improve ACT therapies in patients without the need for lymphodepletion. Lastly, because combining TLR agonists has been reported to further activate the innate immunity, coadministration of these adjuvants and/or coinhibitory blocker with TLR agonists might enhance T cell-based therapy.

## 12. Potentiating Antitumor T Cells: Costimulation, Cytokines, and Microbial Signals

Adoptive T cell transfer therapies mediate potent results in the clinic [93, 136]. Yet, there are still cases of relapse and nonresponders in ACT trials. Thus there is a need to improve how T cells are manipulated* ex vivo* to enhance their* in vivo* antitumor immune responses. A way to generate more therapeutic T cells for ACT is to address how they can be manipulated* ex vivo*. T cells can be manipulated* ex vivo* in several ways: (1) the stimulation process (such as with engineered CD3 beads, artificial antigen presenting cells, or specific peptides); (2) the costimulatory molecules used (ICOS); (3) the polarizing cytokine milieu to generate specific T cell subsets (i.e., Tc1 or Th17); (4) addition of TLR agonists (such as CpG); (5) culturing cells with homeostatic cytokines (IL-15 or IL-21) or pharmaceutical drugs that target the Wnt/*β*-catenin or PI3 kinase signaling pathway.

Beyond how a cell is influenced during differentiation, it is critical to understand an effector cells' memory phenotype and how it might afford long-term protection. Interestingly, the central memory phenotype of the cell has been reported to be better at mediating tumor destruction than effector memory T cells [86]; therefore generating T cells with durable memory or “stemness” characteristics is of interest [137, 138]. Additionally, gut microbes might impact adoptively transferred T cells' memory profile.

## 13. Intestinal Microbiota Affects Adjuvant Therapy (CpG and IL-10 Blockade)

Given the vast diversity of microbial composition among individuals, it is difficult to fully understand all of the potential mechanisms by which the microbiota promotes or abrogates oncogenesis. However, recent studies in murine models have shed light on how the microbiota regulates the immune system in the context of cancer [139, 140]. Specifically, Iida and colleagues found that tumor-bearing mice treated with an immunotherapy consisting of intratumoral CpG ODN injections and IL-10 neutralizing antibodies mediated potent regression of various types of tumors* in vivo*. Interestingly, this potent antitumor response was impaired when the mice were treated with microbiota-depleting antibiotics. Furthermore, they found that tumor-bearing mice failed to respond to oxaliplatin (a chemotherapeutic drug that drives antitumor T cell immunity) when they were depleted of intestinal microbes [141]. Consistent with this work, Viaud and colleagues found that cyclophosphamide, an alkylating chemotherapy agent, impaired the intestinal barrier leading to increased translocation of commensal bacteria and, in turn, augmented the generation of pathogenic Th17 cells and memory Th1 cells that were dependent on the composition of gut microbiota [142]. These findings underscore that the microbiota plays an important role in regulate cancer immunology and immunotherapy.

## 14. CD4**^+^** T Cells for ACT: Microbes Enhance Th17 Cells

Veldhoen and coworkers reported that distinct microbial TLR ligands promote Th17 cell differentiation [143]. Thus, it is not surprising that multiple investigators later found that germ-free or antibiotic-treated animals have fewer Th17 cells than wild-type animals. Indeed, Ivanov and associates first reported that a distinct microbe played a role in the generation of Th17 cells when they discovered that the same strain of C57BL6 mice from one facility (Taconic Laboratories) had dramatically more Th17 cells than the same mouse strain from a different facility (the Jackson Laboratories) [144]. Additional investigation revealed that a single, specific commensal bacterial microbe, called segmented filamentous bacteria (SFB), expressed in Taconic mice was responsible for supporting Th17 cells. In fact, in the absence of SFB (for instance, in germ-free or antibiotic-treated mice deficient in SFB) Th17 cells failed to develop. Interestingly, reconstituting mice with SFB restored Th17 development [145]. This basic finding that SFB promotes Th17 cell generation has significant clinical implications, as SFB-induced Th17 cells worsen the disease pathogenesis of arthritis and diabetes in mice [30, 146]. Conversely, emerging work from Viaud and collaborators suggests that microbial translocation induced by cyclophosphamide, a common chemotherapy used to treat various cancers, enhances the generation of Th1 and Th17 cells in mice, thereby augmenting their capacity to kill tumors [142]. They found that both* L. johnsonii* and* E. hirae* microbes promoted the differentiation of naïve CD4^+^ T cells into memory Th1 and pathogenic Th17 cells* in vitro*, in the presence of bone marrow-derived dendritic cells. Interestingly while* E. Coli*-derived LPS was found to potentiate the antitumor activity of adoptively transferred CD8^+^ T cells [147], they found that microbial LPS did not potentiate the antitumor activity of Th17 cells. Complementary to Littman's lab, they established that association of tumor-bearing germ-free mice with SFB, which promotes Th17 cell differentiation, had a detrimental impact on the growth of sarcoma in CTX-treated mice. Given that adoptively transferred TRP-1 Th17 cells mediate potent regression of melanoma in lymphodepleted mice, it will be important to uncover how these microbes (*L. johnsonii, E. hirae, E. coli*, and SFB) impact Th17 cell-mediated tumor immunity. Additionally, it is unknown whether similar findings will correlate to IL-17-producing CD8^+^ T (Tc17) cells, which have also been shown to have antitumor potential in irradiated mice [148]. Overall, it is clear that particular attention should be paid to a hosts' microflora as the current and posttherapy immune response could be significantly impacted from even minor variations in gut microbe colonization.

## 15. Microbiota-Targeted Therapies to Treat Cancer Patients

Antibiotics used to treat cancer patients after chemotherapy have been reported to differentially impact the degree of autoimmunity in mice with arthritis [30]. More specifically, neomycin was found to worsen rheumatoid arthritis while vancomycin and metronidazole reduced the severity of this disease [30]. Interestingly, these antibiotics differentially impacted the generation of gut-derived Th17 cells, which play a role in regulating immunity to self-tissue. However, the role of antibiotics in regulating T cell-mediated tumor immunity remains incompletely elucidated. Studies have shown that broad-based antibiotic ciprofloxacin can impair the function and antitumor activity of transferred CD8^+^ T cells in irradiated mice [6]. How ciprofloxacin regulates Th17 cells in these mice and man and the composition of the microbiome are still unclear. Based on work by the Mathis lab, we suspect that antibiotics might distinctly regulate T cell-mediated tumor immunity. We posit that different types of antibiotics will differentially regulate Th1, Treg, and Th17 cells* in vivo*, thereby regulating the antitumor capacity of infused CD8^+^ T cells. If true, it is possible that changing the diet of the patient or treating them with certain antibiotics or probiotics would impact their immune system, particularly Th17 cells, thereby improving CD8^+^ T cell-based therapies in the patients.

## 16. Concluding Remarks

Numerous studies underscore the importance of microbes in regulating the innate and adaptive immune response. The potent adjuvant functions of TLRs have been thoroughly investigated for cancer therapies, but the vast amount of data that has accumulated reveals that in oncological settings these therapies lack robust long-term efficacy. We anticipate that the most robust anticancer therapies in the future will involve the use of genetically engineered tumor-specific T cells and checkpoint modulators. It is of note that lymphodepletion, via irradiation, generates an ideal environment for the infused T cells to thrive and regress tumors via depleting suppressive immune cells and activating the innate immune system via microbial translocation. Yet, this preparative regimen can mediate toxic side effects to the patients. Therefore, we have discussed using alternative methods that mimic the beneficial aspects resulting from irradiation. Examples are the delivery of cell depleting antibodies to remove cytokine sinks and regulatory cells, the administration of homeostatic cytokines to enhance cell expansion, and treatment with microbe-based adjuvants to activate the innate immune system. Since Coley's work, we have found that microbes and particularly TLR agonists played a role in shape immunity to cancer. Future studies will benefit by marrying tumor-specific adaptive responses with the innate responses induced via TLR signaling.

## Figures and Tables

**Table 1:** Human TLRs.

Toll-like receptor	Recognized PAMP/DAMP	Localization	Cellular expression	Therapeutic agonist
TLR3	dsRNA	Endosome	Myeloid DC, B cells Intestinal epithelium	Poly(I:C)
TLR4	LPS, heat shock proteins fibronectin, uric acid glycolipids, heparan sulfate	Plasma membrane	MΦ, myeloid DCs, mast cells Intestinal epithelium	LPS, MPL
TLR5	Flagellin	Plasma membrane	MΦ, subset DC Epithelium, granulocytes	Flagellin
TLR7/8	ssRNA	Endosome	MΦ, plasmacytoid DCs mast cells, B cells, myeloid DCs	Imiquimod Resiquimod
TLR9	Unmethylated CpG-rich DNA	Endosome	MΦ, plasmacytoid DCs B cells	Unmethylated CpG

Abbreviations: DAMP, damage-associated molecular pattern; DC, dendritic cell; dsRNA, double-stranded RNA; LPS, lipopolysaccharide; MPL, monophosphoryl lipid A; PAMP, pathogen-associated molecular pattern; Poly(I:C), polyinosinic:polycytidylic acid; ssRNA, single-stranded RNA; TLR, toll-like receptor.
